# ATP-evoked intracellular Ca^2+^ transients shape the ionic permeability of human microglia from epileptic temporal cortex

**DOI:** 10.1186/s12974-021-02096-0

**Published:** 2021-02-15

**Authors:** Nicole Piera Palomba, Katiuscia Martinello, Germana Cocozza, Sara Casciato, Addolorata Mascia, Giancarlo Di Gennaro, Roberta Morace, Vincenzo Esposito, Heike Wulff, Cristina Limatola, Sergio Fucile

**Affiliations:** 1 IRCCS Neuromed, Pozzilli, IS Italy; 2 Department of Human Neurosciences, Sapienza Rome University, Rome, Italy; 3 Department of Pharmacology, University of California, Davis, CA USA; 4 Department of Physiology and Pharmacology “V. Erspamer”, Sapienza Rome University, Rome, Italy

**Keywords:** Temporal lobe epilepsy, K_Ca_3.1, Neuroinflammation, Purinergic signaling, Primary cultures, Perforated patch

## Abstract

**Background:**

Intracellular Ca^2+^ modulates several microglial activities, such as proliferation, migration, phagocytosis, and inflammatory mediator secretion. Extracellular ATP, the levels of which significantly change during epileptic seizures, activates specific receptors leading to an increase of intracellular free Ca^2+^ concentration ([Ca^2+^]_i_). Here, we aimed to functionally characterize human microglia obtained from cortices of subjects with temporal lobe epilepsy, focusing on the Ca^2+^-mediated response triggered by purinergic signaling.

**Methods:**

Fura-2 based fluorescence microscopy was used to measure [Ca^2+^]_i_ in primary cultures of human microglial cells obtained from surgical specimens. The perforated patch-clamp technique, which preserves the cytoplasmic milieu, was used to measure ATP-evoked Ca^2+^-dependent whole-cell currents.

**Results:**

In human microglia extracellular ATP evoked [Ca^2+^]_i_ increases depend on Ca^2+^ entry from the extracellular space and on Ca^2+^ mobilization from intracellular compartments. Extracellular ATP also induced a transient fivefold potentiation of the total transmembrane current, which was completely abolished when [Ca^2+^]_i_ increases were prevented by removing external Ca^2+^ and using an intracellular Ca^2+^ chelator. TRAM-34, a selective K_Ca_3.1 blocker, significantly reduced the ATP-induced current potentiation but did not abolish it. The removal of external Cl^−^ in the presence of TRAM-34 further lowered the ATP-evoked effect. A direct comparison between the ATP-evoked mean current potentiation and mean Ca^2+^ transient amplitude revealed a linear correlation. Treatment of microglial cells with LPS for 48 h did not prevent the ATP-induced Ca^2+^ mobilization but completely abolished the ATP-mediated current potentiation. The absence of the Ca^2+^-evoked K^+^ current led to a less sustained ATP-evoked Ca^2+^ entry, as shown by the faster Ca^2+^ transient kinetics observed in LPS-treated microglia.

**Conclusions:**

Our study confirms a functional role for K_Ca_3.1 channels in human microglia, linking ATP-evoked Ca^2+^ transients to changes in membrane conductance, with an inflammation-dependent mechanism, and suggests that during brain inflammation the K_Ca_3.1-mediated microglial response to purinergic signaling may be reduced.

**Supplementary Information:**

The online version contains supplementary material available at 10.1186/s12974-021-02096-0.

## Introduction

Epilepsy is a neurological disorder characterized by an altered balance between neuronal excitation and inhibition, involving profound changes in the function of brain networks and cells, including microglia [1]. Microglia are the resident immune cells of the central nervous system (CNS), where they play a homeostatic role of surveillance [2], contributing to synaptic pruning and neuromodulation [3, 4]. These key functions are regulated by the interaction of microglia with several modulatory molecules, including neurotransmitters, neuropeptides, and cytokines [5–7]. An important modulator of microglial functions is extracellular ATP [8], which is significantly altered during epileptic seizures [9]. Extracellular ATP activates specific ionotropic and metabotropic purinergic receptors on the microglial membrane, leading to an increase of intracellular free Ca^2+^ concentration ([Ca^2+^]_i_) [10]. Ca^2+^ is a key transducer modulating several microglial activities, such as proliferation, migration, phagocytosis, and inflammatory mediator secretion [11, 12]. In particular, changes in [Ca^2+^]_i_ rapidly tune the membrane ionic conductance [13] which, in turn, modulates relevant microglial function, such as motility, phagocytosis, and generation of ROS and cytokines [14]. Given that Ca^2+^ activates membrane channels mainly through the interaction with diffusible mediators, such as cGMP [15], possible problems arise from the washout of crucial cytoplasmic diffusible factors in the usual conditions of whole-cell recordings [16, 17]. To avoid the loss of intracellular mediators of Ca^2+^ activity, we here applied the perforated patch-clamp technique to the quantitative analysis of the ionic conductance induced by ATP-evoked Ca^2+^ transients in human microglia isolated from the temporal cerebral cortices of adult epileptic patients. We identified the specific channels involved in the response to [Ca^2+^]_i_ increase before and after stimulation with LPS and confirmed a primary role for the intermediate-conductance Ca^2+^-activated K_Ca_3.1 channel [18, 19].

Our results are discussed in light of the modulation of K_Ca_3.1 activity as a tool to modulate microglia-induced neuroinflammation in epileptic patients [20].

## Methods

### Patients

Surgical specimens were obtained from the temporal neocortex of 13 subjects with drug-resistant temporal lobe epilepsy (Table S1) operated on at the Epilepsy Surgery Center of IRCCS Neuromed, Pozzilli (IS), Italy. Informed consent was obtained from each patient to use part of the surgically resected material for experiments, and the local ethics committee approved the selection processes and procedures (approval number 5/2019).

### Isolation of primary human microglia

The isolation procedure was as described by Rustenhoven et al. [21]. Following surgical resection, tissue was transported to the research facility in cold HBSS in less than 20 min. Approximately 1-2 g of tissue was washed in HBSS (Gibco), and meninges and visible blood vessels were removed. Tissue was diced into pieces approximately 1 mm^3^ using a sterile scalpel and transferred to a 50-mL falcon tube containing 10 mL enzyme dissociation mix (10 U/mL DNase (Invitrogen) and 2.5 U/mL papain (Worthington) in Hibernate-A medium (Gibco)) per gram of tissue for 10 min in an incubator at 37 °C with gentle rotation. The tissue was then gently triturated and returned to the incubator for another 10 min. Dissociation was slowed by adding an equal volume of Dulbecco’s modified eagle medium: Nutrient mixture F-12 (DMEM/F12 with 1% FBS (Gibco). The cell suspension was passed through a 70 μm cell strainer (Becton Dickinson) to enhance culture homogeneity. Cells were centrifuged at 160×*g* for 10 min and resuspended in 20 mL of media containing DMEM/F12 with 1% FBS, 1% GlutaMAX (Gibco), 1% penicillin-streptomycin-glutamine (PSG). The cell suspension was transferred to Petri dishes and incubated overnight at 37 °C with 95% air/5% CO_2_. The following day non-adherent or loosely adherent cells were removed. The adherent cells were washed twice, and culture media were added (DMEM/F12 with 10% FBS and 1% PSG). Cells were used for functional studies after at least 5 days in culture. Human microglial cells were activated by adding LPS (100 ng/ml) to the culture medium 48 h before the experiments [22, 23].

### Immunocytochemistry

Cells were fixed in 4% paraformaldehyde (ChemCruz) for 15 min and washed in PBS. After cell permeabilization with 0.2% Triton X-in PBS, cells were blocked (1% BSA in PBS) for 1 h, at RT, and incubated overnight at 4 °C with Iba1 and GFAP antibody (1:700; Wako) diluted in PBS with 0.1% BSA. Cells were washed three times in PBS and incubated with the fluorophore-conjugated secondary antibodies (1:2000, Catalog#A11012, A-21202, Invitrogen) for 45 min. After three washes in PBS, nuclei were counterstained with Hoechst 33258 for 5 min and cells washed in PBS. Coverslips were mounted on dishes using the Fluorescent Mounting Medium (DakoCytomation). Images were digitized using a CoolSNAP camera (Photometrics) coupled to an ECLIPSE Ti-S microscope (Nikon) and processed using MetaMorph 7.6.5.0 image analysis software (Molecular Device).

### Patch-clamp experiments

Currents were recorded with a Multiclamp 700B amplifier and Clampex 10.5 software (Molecular Devices) in the perforated patch-clamp configuration to reduce the perturbation of the intracellular milieu, by using escin (50 μM). Patch-clamp recordings were obtained using borosilicate glass electrodes (4-6 MΩ) filled with different intracellular solutions: in the perforated patch or normal whole-cell configuration, in mM, K-gluconate 140, HEPES 10, BAPTA 5, MgCl_2_ 2, and Mg-ATP 2, pH 7.3, adjusted with KOH; in normal whole-cell configuration, with 1 μM intracellular Ca^2+^, in mM, K-gluconate 145, HEPES 10, EGTA 10, CaCl_2_ 8.5, MgCl_2_ 2, pH 7.3, adjusted with KOH. During recordings, cells were continuously perfused using normal external solutions (NES), containing, in mM, NaCl 140, HEPES 10, KCl 2.8, CaCl_2_ 2, MgCl_2_ 2, glucose 10, pH 7.3 adjusted with NaOH. In some experiments a low Cl^−^ external solution was used, containing, in mM, Na-gluconate 140, HEPES 10, KCl 2.8, CaCl_2_ 2, MgCl_2_ 2, glucose 10. Cells were held at −70 mV and currents were elicited with voltage ramps (from −120 mV to +40 mV, 200 ms) every 2 s. The current density at −20 mV was determined as the current amplitude/cell capacitance (pA/pF). Cell capacitance was continuously monitored. To avoid Ca^2+^ entry in the intracellular compartment, in selected experiments, a Ca^2+^-free external solution was used, containing, in mM, NaCl 140, HEPES 10, KCl 2.8, EGTA 2, MgCl_2_ 4, and glucose 10 (pH=7.3).

### Ca^2+^-transient recordings

Changes in free intracellular Ca^2+^ concentration ([Ca^2+^]_i_) were measured by time-resolved digital fluorescence microscopy, using an integrated acquisition system (Scientifica), composed of an upright reflected fluorescence microscope (Olympus) and the Optofluor software (Molecular Devices). Cells were incubated with the Ca^2+^ indicator Fura-2 acetoxylmethylester (1 μM) for 45 min at 37 °C in culture medium. The changes of [Ca^2+^]_i_ were expressed as R = F340/F380, where F340 and F380 are the fluorescence intensities measured from individual cells at an emission wavelength of 510 nm illuminating the specimen with 340 and 380 nm excitation wavelengths, respectively. Cells were continuously perfused during the experiment. The acquisition frequency was 0.33 Hz.

### Data and statistical analysis

Data sampling was performed using the pClamp 10 software (Molecular devices) and the OptoFluor (CAIRN) software. Data analysis was performed using SigmaPlot 14.0 (Systat). All data are expressed as means ± standard error mean and analyzed using paired *t* test or one-way ANOVA, as appropriate. Significance for all tests was set at *p* < 0.05, with statistical power > 0.8. For each experiment, data were pooled from microglial cells obtained from the patients indicated in the corresponding figure legends. The linear regression is calculated by the SigmaPlot software by the least-squares method.

## Results

**Fig. 1:**
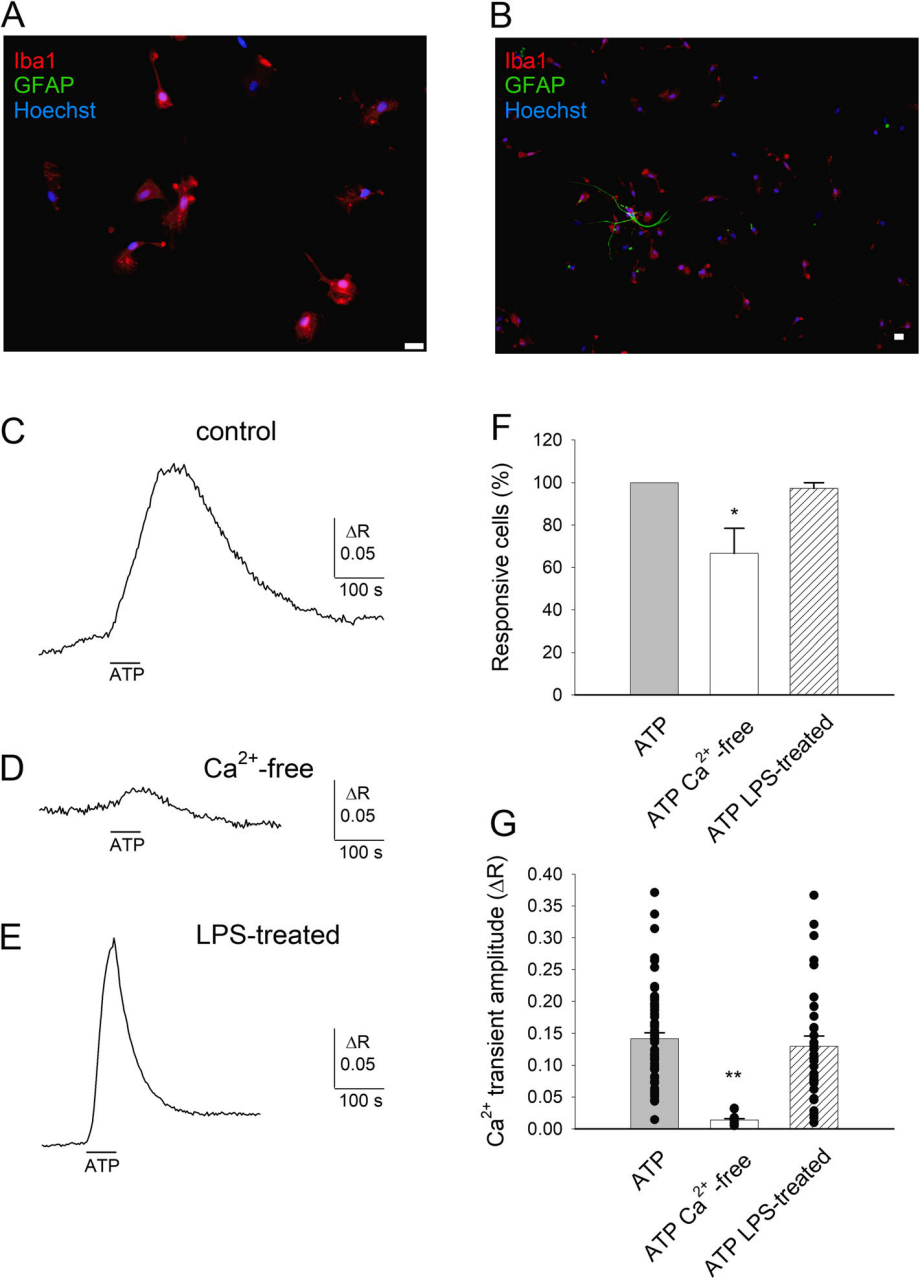
Extracellular ATP evokes Ca^2+^ entry in highly pure primary cultures of human microglia. **a**, **b** Representative immunofluorescence images of primary cultures of human microglial cells (patient #12) marked with Iba1 and GFAP antibody (red and green, respectively) and nuclei marked with Hoechst 33258 (blue). Horizontal bar = 20 μm. Please note in **b** the presence of a single astrocyte. **c** Typical time-course of [Ca^2+^]_i_ changes elicited by ATP application (100 μM, 1 min, horizontal line) in a single human microglial cell (#9). **d** Typical time-course of [Ca^2+^]_i_ changes elicited by ATP application in the absence of external Ca^2+^ (10 min preincubation; #10). **e** Typical time-course of [Ca^2+^]_i_ changes elicited by ATP application in a human microglial cell pretreated with LPS for 48 h (#12). **f** Histogram representing the percentage of microglial cells responding to ATP in control conditions (62 out 62 cells, 16 optical fields, #9), in Ca^2+^-free medium (15 out 23 cells, 4 optical fields, #10; one asterisk denotes significantly different from control, *p*=0.023) or after LPS treatment (41 out 42 cells, 6 optical fields, #12). **g** Histogram representing the mean amplitude of ATP-induced Ca^2+^ elevations, same cells as **c**, **d**, and **e**. Two asterisks denote significantly different from both other values, *p*<0.001. Black circles represent individual values

Human microglial cells play an important role in controlling the inflammatory status of the brain in epileptic patients [1]. To better define their functional role, we aimed to describe the molecular pathways activated in human microglia by activity-driven mechanisms, such as purinergic signaling [9, 24]. Thus, we established primary cultures of human microglia derived from the temporal cortex surgically resected from patients with temporal lobe epilepsy. These cultures were 98.8±1.2 % positive for Iba1 (Fig. 1a, b; *n*=7). The residual cells were contaminating astrocytes, as identified by GFAP staining (Fig. 1b). First, to study the functional relationship between Ca^2+^ mobilization and the modulation of membrane conductance in human microglia, we quantified the Ca^2+^ signaling elicited upon ATP stimulation [8]. We chose an extracellular ATP concentration of 100 μM based on two considerations: (i) the concentration should be able to elicit clear Ca^2+^ transients in microglia [25]; (ii) the increase of ATP concentration is limited during seizures [24] and never reaches the millimolar range necessary to activate P2X7 receptors [26]. As expected, all microglial cells responded to the application of ATP (100 μM) with a clear increase of [Ca^2+^]_i_ (Fig. 1c, f, g; Table 1). The ATP-induced Ca^2+^ mobilization was dependent on Ca^2+^ entry from the extracellular space but also from intracellular compartments, as indicated by the smaller [Ca^2+^]_i_ increase observed upon removal of external Ca^2+^ (Fig. 1d, f, g; Table 1). Under these conditions, the Ca^2+^ transients had a smaller amplitude and a visible Ca^2+^ response was observed in a reduced percentage of cells (65.2%; Fig. 1f), likely because the signals were close to the detection limit of the essay. Thapsigargin, a selective blocker of endoplasmic reticulum Ca^2+^ ATPases [30], evoked small-amplitude Ca^2+^ transients in a small percentage of cells (Table 1), confirming that in our preparation, the amount of Ca^2+^ releasable from intracellular compartments is limited. To simulate the effects of an inflammatory stimulus, cells were preincubated with LPS for 48 h [22, 23]. Under these conditions, ATP-evoked Ca^2+^ transients exhibited similar amplitudes (Fig. 1e, g) but faster kinetics than in unstimulated cells: both rise time and half decay time were significantly shorter than in untreated cells (Fig. 1e; Table 1).

**Table 1:** Properties of Ca^2+^ transients evoked in human microglial cells under different experimental conditions

Stimulus	Resp. cells (%)	Basal (R)	Peak (R)	Δ*R*	*T*_0-100_ (s)	*T*_100-50_ (s) [*n*]
ATP	100 (62/62)	0.22±0.01	0.36±0.02	0.142±0.002	114±8	419±24 [27]
ATP (LPS)	97.6 (41/42)	0.219±0.007	0.35±0.02	0.13±0.02	67±6	353±24 [28]
ATP Ca^2+^-free	65.2 (15/23)	0.177±0.007	0.191±0.008	0.014±0.004	336±29	Nd [15]
GSK 1702934A	61.6 (74/120)	0.42±0.01	0.48±0.02	0.059±0.004	42±3	441±19 [29]
Thapsigargin	28.4 (19/67)	0.33±0.03	0.37±0.04	0.039±0.004	47±6	307±34 [5]

Amplitude and kinetic parameters of evoked Ca^2+^ transients were measured on a subset [*n*] of total responsive cells, in which it was possible to resolve the peak and the recovery of the Ca^2+^ signal. Data were obtained from 3, 2, 3, 2, and 2 different patients for ATP, ATP in LPS-treated cells, ATP in Ca^2+^-free, GSK 1702934A, and thapsigargin, respectively

**Fig. 2:**
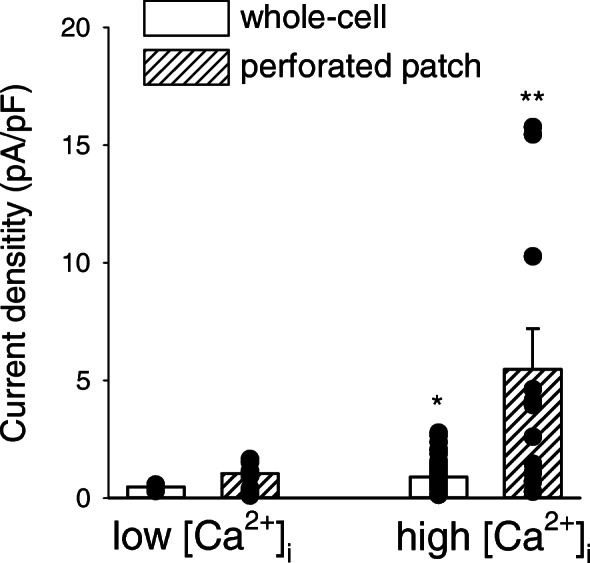
Intracellular dialysis limits the Ca^2+^-induced potentiation of the whole-cell transmembrane currents recorded from human microglia. Histogram representing the mean whole-cell current densities recorded from human microglial cells in unstimulated conditions or in high [Ca^2+^]_i_ conditions, as indicated, with or without internal dialysis (white or hatched bars, respectively). High [Ca^2+^]_i_ condition was obtained by using a controlled intracellular solution containing 1 μM free Ca^2+^. Black circles represent individual cells. High [Ca^2+^]_i_ values significantly higher than respective low [Ca^2+^]_i_ values. ANOVA, *p=0.023; **p<0.001. Number of cells, from left to right, 4 (patient #12), 13 (6 cells from #3, 5 from #9, and 2 from #12), 51 (9 cells from patient #1, 17 from #2, 6 from #5, and 19 from #13), 13 (6 cells from #3, 5 from #9, and 2 from #12), respectively

**Fig. 3:**
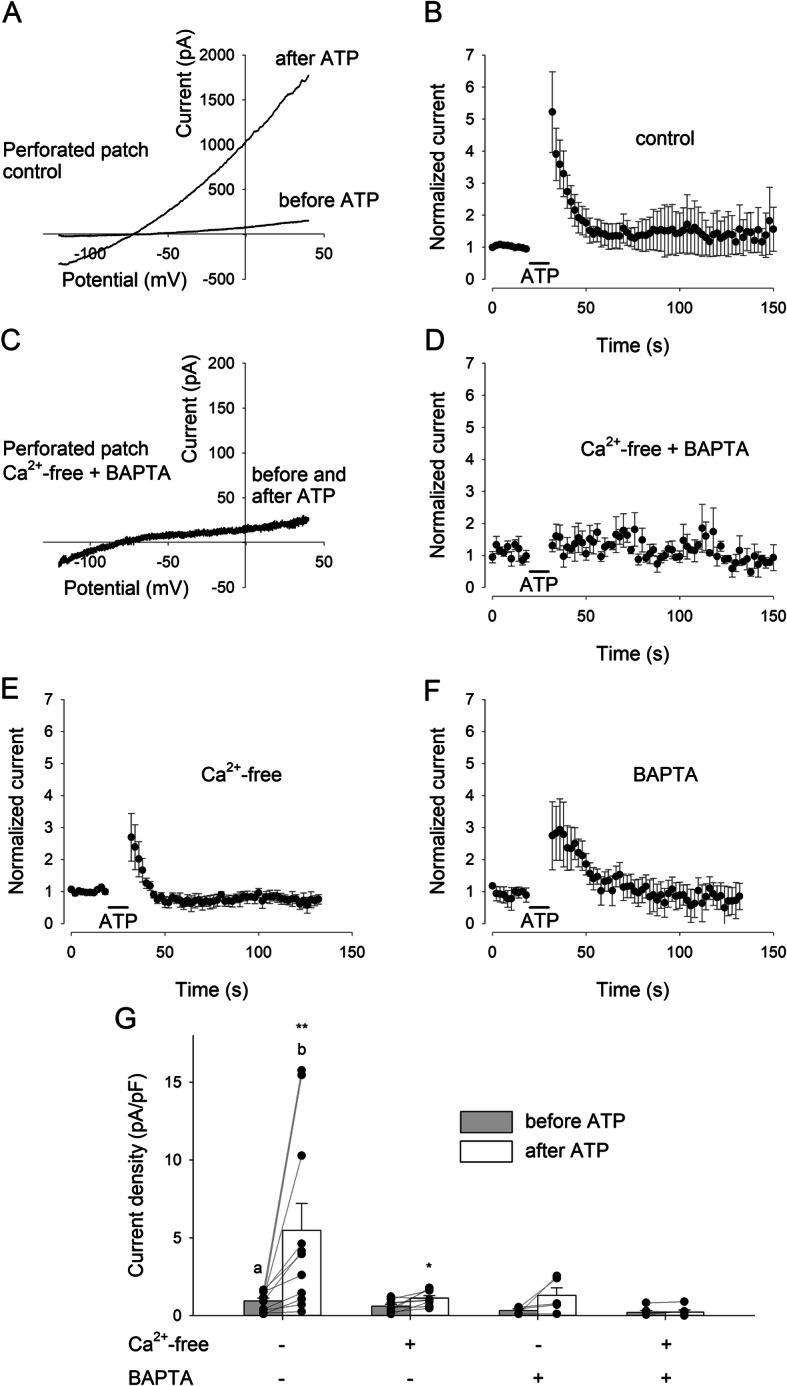
The transient ATP-induced potentiation of whole-cell currents in human microglia depends on the [Ca^2+^]_i_ increase. **a** Typical I-V relation obtained by applying a single voltage ramp (from −120 mV to +40 mV, 200 ms) before and after the administration of ATP (100 μM, 10 s), on an representative human microglial cell (patient #3) out of the 11 cells recorded in the same conditions. Whole-cell currents were recorded in the voltage-clamp configuration using the perforated patch technique, to avoid cell dialysis. Please note the strong increase in the outward current after ATP. **b** Time course of the mean current amplitude measured at −20 mV in control conditions, averaged from 11 cells (6 from #3 and 5 from #9). Voltage ramps were applied every 2 s. Current amplitudes were normalized to the value averaged from the 5 currents preceding ATP administration. **c** Typical I-V relation obtained by applying a single voltage ramp before and after the administration of ATP, on a representative cell (#7) out of the 6 cells recorded in the same conditions. **d** Time course of the mean current amplitude measured at −20 mV in the absence of external Ca^2+^ in cells preincubated with the Ca^2+^ chelator BAPTA-AM (20 μM), averaged from 6 cells (#7). Please note that ATP-induced current potentiation is abolished if [Ca^2+^]_i_ increase is prevented. **e** Time course of the mean current amplitude measured at −20 mV in the absence of external Ca^2+^, averaged from 9 cells (#10). **f** Time course of the mean current amplitude measured at −20 mV in cells preincubated with the Ca^2+^ chelator BAPTA-AM (20 μM), averaged from 5 cells (#4). **g** Histogram representing the mean current density measured at −20 mV immediately before (gray) and after (white) the ATP administration, in different experimental conditions, as indicated. Please note that in the absence of extracellular Ca^2+^ cells preincubated with BAPTA-AM shows a reduction of basal current density along with the complete lack of ATP-induced current potentiation. The numbers of recorded cells for control (left bars), Ca^2+^-free, only BAPTA, and Ca^2+^-free + BAPTA experiments (right bars) were 13 (6 from #3, 5 from #9 and 2 from #12), 9 (#10), 5 (#4), and 6 (#7), respectively. Two asterisks denote significantly higher than before ATP, *p*<0.001. *p=0.025. **a** Mean basal value in control condition is significantly higher than in BAPTA (*p*=0.033) and in Ca^2+^-free + BAPTA (*p*=0.007). **b** Mean peak value in control condition is significantly higher than in Ca^2+^-free (*p*=0.020), in BAPTA (*p*=0.025), and in Ca^2+^-free + BAPTA (*p*=0.006)

The study of the Ca^2+^-dependent regulation of ionic conductances in resting and LPS-treated human microglia strictly relies on good preservation of diffusible cytoplasm factors. Thus, whole-cell transmembrane current recordings were performed in the perforated patch-clamp configuration, avoiding cell dialysis [31]. A direct comparison showed that in the presence of high [Ca^2+^]_i_ (1 μM) the current amplitudes recorded from dialyzed cells are much smaller than those recorded from intact cells with high [Ca^2+^]_i_ and are not significantly different from intact cells at rest (Fig. 2), confirming that the wash-out of intracellular factors, likely channel-activating Ca^2+^-binding molecules, prevents a complete current potentiation in response to [Ca^2+^]_i_ increase [15]. Under perforated patch conditions, during a stimulation protocol consisting of voltage ramps from −120 mV to +40 mV, applied every 2 s, the administration of ATP (100 μM) to human microglia induces a clear increase of the transmembrane current, as exemplified in Fig. 3a. Quantified at a membrane potential of −20 mV, this current potentiation was transient (Fig. 3b) and was completely abolished when [Ca^2+^]_i_ increase was prevented by removing external Ca^2+^ and loading cells with the Ca^2+^ chelator BAPTA-AM (20 μM, Fig. 3c, d). Following ATP application, the current density measured at −20 mV changed from a mean basal value of 0.9±0.2 pA/pF to a mean peak value of 4.8±1.5 pA/pF (Fig. 3g; *n*=13). In the absence of external Ca^2+^ (Fig. 3e) or in cells preincubated with BAPTA-AM (Fig. 3f), the mean basal current density was reduced, as expected, to 0.6±0.1 pA/pF and 0.33±0.09 pA/pF (Fig. 3g; *n*=9 and *n*=5), respectively, with an ATP-induced effect that was significant only in Ca^2+^-free conditions (1.1±0.2 pA/pF). In Ca^2+^-free plus BAPTA conditions, the measured current density did not change from the basal value of 0.2±0.1 pA/pF. These results indicate that the ATP-mediated modulation of human microglial transmembrane currents depends on the ability of the cells to mobilize intracellular Ca^2+^.

**Fig. 4:**
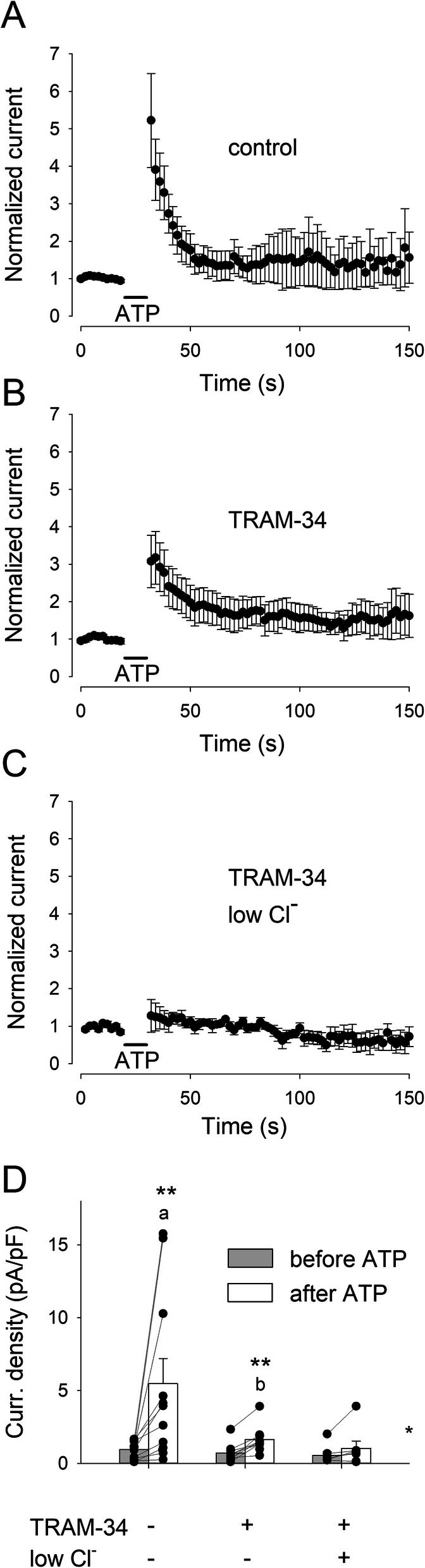
The ATP-induced current potentiation is due to Ca^2+^-dependent K^+^ and Cl^−^ conductances. **a** Time course of the mean current amplitude measured at −20 mV in control conditions, averaged from 11 cells (6 from #3 and 5 from #9; same data of Fig. 3b). Voltage ramps were applied every 2 s. Current amplitudes were normalized to the value averaged from the 5 currents preceding ATP administration. **b** Time course of the mean current amplitude measured at −20 mV in the presence of TRAM-34 (1 μM), averaged from 10 cells (#3). **c** Time course of the mean current amplitude measured at −20 mV in the absence of external Cl^−^ and in the presence of TRAM-34 (1 μM), averaged from 7 cells (#11). **d** Histogram representing the mean current density measured at −20 mV immediately before (gray) and after (white) the ATP administration, in different experimental conditions, as indicated. The numbers of recorded cells for control (left bars), only TRAM-34, and Cl^−^-free + TRAM-34 (right bars) were 13 (6 from #3, 5 from #9, and 2 from #12), 10 (#3), and 7 (#11), respectively. Two asterisks denote significantly higher than before ATP, *p*<0.001. *p=0.031. **a** Mean peak value in control condition is significantly higher than in Cl^−^-free + TRAM-34 (*p*=0.016). **b** Mean peak value in TRAM-34 is significantly higher than in Cl^−^-free + TRAM-34 (*p*=0.040)

**Fig. 5:**
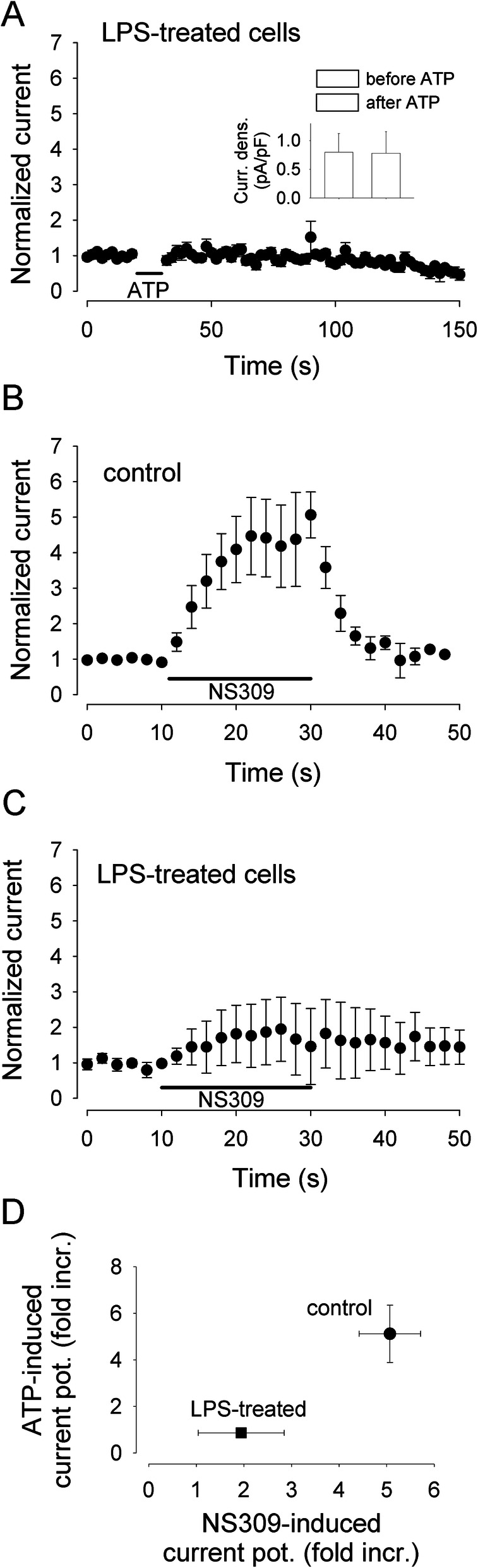
The ATP-induced current potentiation is downregulated in LPS-treated human microglia. **a** Time course of the mean current amplitude measured at −20 mV in control conditions, averaged from 5 cells (3 from #10 and 2 from #12) pre-treated with LPS (100 ng/ml for 48 h; LPS absent during the recording). Please note that LPS treatment completely abolished the ATP-induced current potentiation (*p*=0.027 when compared with control cells; *n*=5). **b** Time course of the mean current amplitude measured at −20 mV during the application of the selective K_Ca_3.1 channel activator NS309 (500 nM, 20 s), averaged from 10 cells (2 from #5, 6 from #1, and 2 from #2). Voltage ramps applied every 2 s. Current amplitudes were normalized to the value averaged from the 5 currents preceding ATP administration. **c** Time course of the mean current amplitude during the application of NS309 on LPS-treated cells, averaged from 5 cells (#12). Please note that LPS treatment strongly reduced the NS309 effect. **d** Correlation plot linking the Ca^2+^-mediated ATP-induced current potentiation to the Ca^2+^-independent current potentiation induced by the selective K_Ca_3.1 activator NS309. Data represent mean values ± standard error mean. Bidirectional errors are shown, sometimes not visible due to error amplitude smaller than symbol dimensions. Please note that LPS treatment reduces both types of modulation, indicating a reduction in the functional expression of K_Ca_3.1 as the cause of the lack of Ca^2+^-mediated current potentiation observed in LPS-treated cells

**Fig. 6:**
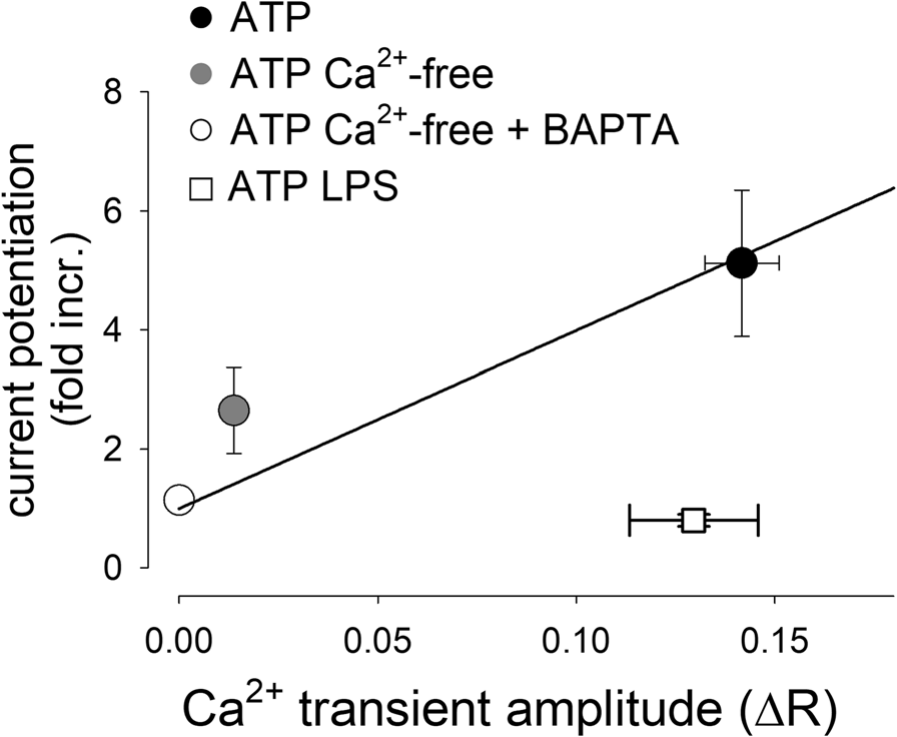
LPS-mediated microglia activation uncouples transmembrane currents from [Ca^2+^]_i_ by reducing the functional expression of K_Ca_3.1. Correlation plot linking the current potentiation to the Ca^2+^ transient amplitudes evoked by ATP in different experimental conditions. The solid line represents a linear regression constrained to pass through the point identified by the coordinates (0, 1). Please note that LPS treatment inhibits the current potentiation leaving unaltered the ability of ATP to increase [Ca^2+^]_i_. Data represent mean values ± standard error mean. Bidirectional errors are shown, sometimes not visible due to error amplitude smaller than symbol dimensions

To dissect the molecular components of the Ca^2+^-dependent currents, we first blocked the K_Ca_3.1 channels, which were previously reported to constitute the major K^+^ conductance in microglia from epileptic patients [2]. The selective K_Ca_3.1 blocker TRAM-34 significantly reduced the mean peak value of the ATP-induced current potentiation (Fig. 4a, b), but did not completely abolish it. In the presence of TRAM-34, ATP potentiated the mean current density from 0.7±0.2 pA/pF to 1.6±0.3 pA/pF (Fig. 4d; *n*=10; *p*<0.001). The removal of external Cl^−^ in the presence of TRAM-34 further lowered the ATP-evoked potentiation (Fig. 4c), which however was still significant: from 0.5±0.3 pA/pF to 1.0±0.5 pA/pF (Fig. 4d; *n*=7; *p*=0.031). This finding confirms the presence of a Ca^2+^-dependent Cl^−^ conductance, previously described in mice [28] also in human microglia. In contrast to what we observed with ATP-induced Ca^2+^ mobilization, the treatment of microglial cells with LPS for 48 h completely abolished the ATP-mediated current potentiation (Fig. 5a). This result, unexpected based on previous studies [19], indicated a functional uncoupling between [Ca^2+^]_i_ increase and current potentiation and led us to analyze the functional expression of K_Ca_3.1 channels upon LPS treatment. Towards this goal, we applied the selective K_Ca_3.1 channel activator NS309 (500 nM) to microglial cells: in unstimulated control cells, NS309 produced a transient fivefold increase in the current measured at −20 mV (Fig. 5b, current density from 0.98±0.02 to 5.1±0.1 pA/pF; *n*=7). This effect was significantly inhibited in LPS-treated cells, but not abolished (Fig. 5c, current density from 0.98±0.09 to 1.9±0.9 pA/pF; *n*=5). LPS treatment similarly decreases both the ATP- and NS309-mediated current potentiation (Fig. 5d), strongly suggesting that LPS incubation, in our conditions, causes a reduction of K_Ca_3.1 function. A direct comparison shows a good correlation between the ATP-evoked mean current potentiation and mean Ca^2+^ transient amplitude, lacking in LPS-treated human microglia (Fig. 6).

To verify whether the mechanism linking Ca^2+^ mobilization and current modulation can be activated by other cellular pathways, we aimed to elicit intracellular Ca^2+^ transients independently from ATP signaling. We, therefore, applied cholinergic agonists previously described to increase intracellular Ca^2+^ in microglia (acetylcholine 100 μM or nicotine 5 μM [12, 32]), but in our hands, the cholinergic stimulation was never able to elicit a Ca^2+^ signal or exert any effect on transmembrane currents (not shown). We then focused on store-operated Ca^2+^ entry, a TRPC-dependent mechanism described in microglia [33]. We applied the selective TRPC3/6 channel activator GSK 1702934 (3 μM) to microglia and measured the [Ca^2+^]_i_ changes as well as the current modulation. This molecule evoked Ca^2+^ transients (Fig. S1A) and caused small but significant current potentiation (Fig. S1B), confirming that Ca^2+^ mobilized independently from purinergic signaling can affect microglial conductances.

## Discussion

Neuroinflammation alters the physiological function of the CNS by modifying the activity of both glia and neurons and the molecular pattern of the extracellular milieu, with significant variation of several mediators such as cytokines, chemokines, and neurotransmitters. Neuroinflammation is a key factor in epileptogenesis [34, 35]. In particular, an inflammatory phenotype of microglial cells has been linked to neurotoxic processes associated with epileptic seizures [1] as well as with epileptogenesis [36]. Microglia are deeply involved in epileptic processes (for review see [1, 37]). In mice, microglia depletion enhances seizure severity and neuronal damage [38], while phenotypic microglia alterations due to elevated mTOR signaling lead to reduced synapse density, moderate neuronal degeneration, and astrocyte proliferation, resulting in severe spontaneous recurrent seizures [39]. Furthermore, aberrant microglia-dependent synaptic pruning in the epileptic brain causes synaptic excitatory/inhibitory imbalance, promoting the progression of epilepsy [40].

Our results on primary microglia from the human epileptic temporal cortex contribute to better define the complex picture of microglial functions in epileptic patients. Even if, in general, studies on human CNS cells suffer from the scarcity of proper controls, they provide crucial information on the molecular and cellular pathways that could be targeted in human cells.

Specifically, our working hypothesis is that the acute response of microglia to extracellular ATP, a relevant mediator released during seizures [24], may be modulated to decrease the pathogenic pro-inflammatory potential of microglia. We confirmed that human microglia respond to extracellular ATP mobilizing intracellular Ca^2+^ and increased Ca^2+^ entry [15]. These cells express different purinergic receptors [4], such as the Ca^2+^-permeable P2X7 and P2X4 ionotropic receptors and the Ca^2+^-mobilizing P2Y metabotropic receptors [29, 41, 42]. P2Y1, P2Y12, and P2Y13 receptors participate in process movement in microglia from epileptic patients [29], and an increase of seizures has been observed in mice lacking the P2Y12 receptor [43]. However, ATP is a weak partial agonist of both P2Y12 and P2Y13 receptors, which are fully activated by ADP [44]. Furthermore, we show that the block of endoplasmic reticulum Ca^2+^ ATPases by thapsigargin evoked very small Ca^2+^ mobilization. Pulled together, these observations suggest that in human microglia from epileptic patients the primary source of ATP-induced [Ca^2+^]_i_ increase is Ca^2+^ entry through P2X ionotropic receptors. Upregulation of both P2X7 and P2X4 receptors has been observed in microglia following experimental seizures [45–47]. The ATP concentration used in the present study (100 μM), not able to activate the low-affinity P2X7 receptors (EC_50_ in the millimolar range) [26], suggests that in our experiments ATP-induced Ca^2+^ entry is primarily mediated by P2X4 receptors, confirming previous results in mouse microglia [42].

The transient increase of [Ca^2+^]_i_ is an essential step in microglial activation, modulating genetic transcription, motility, secretion, and other processes [11]. Furthermore, microglial calcium signaling is altered in awake mice following status epilepticus, indicating calcium as a key second messenger in microglia during epileptogenesis [27]. Ca^2+^ activates several ion channels that affect microglial membrane potential [19]. Extracellular ATP potentiates the transmembrane currents measured in human microglia in a Ca^2+^-dependent manner. However, this effect is completely abolished when [Ca^2+^]_i_ increases are inhibited by chelating Ca^2+^. When we further dissected ionic components contributing to these Ca^2+^-evoked currents, we found both a Cl^−^ and a K^+^ component. A Ca^2+^-dependent Cl^−^ conductance has been recently described in mouse hippocampus [28], but its physiological role needs to be studied in more detail in human cells. Here, we focused on the study of the K^+^ component of the Ca^2+^ dependent conductance. Adult human microglia express different K^+^ channels [19]. Our results confirm that the main channel responsible for the Ca^2+^-induced K^+^ current is K_Ca_3.1 [19], as clearly established by the sensitivity of the current to TRAM-34 [48]. The intermediate-conductance K_Ca_3.1 channel mediates Ca^2+^-induced hyperpolarization and is expressed by different cell types, including erythrocytes [49], leukocytes [50], vascular endothelium [51], bronchial epithelium [52], fibroblasts [53], and other cellular systems (for review see [54]). Consequently, K_Ca_3.1 has been involved in many pathologies, including cardiovascular and respiratory disease [55, 56], diabetes [57], and tumor pathogenesis [58]. Several reports highlighted the pathological role of K_Ca_3.1 in brain tumors, with a special focus on glioblastoma [59]. Many studies have identified K_Ca_3.1 as a relevant therapeutical target, and several pharmacological tools have been developed [54], which could be helpful in different pathological contexts, including epilepsy [60].

The observed ATP-induced current potentiation was transient and was quantitatively correlated to [Ca^2+^]_i_ increase. The key role of Ca^2+^ is confirmed by the fact that a small [Ca^2+^]_i_ increase due to a different stimulus, the activation of store-activated Ca^2+^ entry mediated by TRPC3/6 [33], is still able to potentiate the microglial conductance. We also investigated the possibility that cholinergic stimulation could elicit Ca^2+^ transients [32, 48], therefore, affecting microglial conductance, but in our experimental conditions, neither acetylcholine nor nicotine evoked a signal. These different results may arise from dissimilar conditions (species, pathology, age, culture conditions) and would require further investigation.

The activation of K_Ca_3.1 by Ca^2+^ is mediated by calmodulin [61], which is constitutively bound to the C-terminus of the channels [62] and can be modulated by other intracellular soluble factors, such as ATP or PKA [63–65]. For this reason, an intact cytoplasm is needed to study the modulation of Ca^2+^ dependent conductances. Thus, the best experimental approach to analyze the function of microglial conductances is the perforated patch-clamp technique, avoiding intracellular dialysis [31]. To the best of our knowledge, our study is the first to apply perforated patch to adult human microglia. The complete or partial washout of diffusible factors during conventional whole-cell electrophysiological recording may affect the amplitudes, the kinetics, and the modulation properties of the recorded currents, leading to a profound alteration of the observed effects. These methodological considerations are particularly relevant when comparing microglial conductances in different conditions.

We used LPS treatment to investigate microglia response to ATP in an inflammatory environment. Detection of LPS in the brain is mainly carried out by Toll-like receptor 4, mostly expressed by microglia [66]. Selective microglial ablation in the dorsal hippocampus highlighted a neuroprotective role of microglia following a 24-h LPS treatment in the pilocarpine seizure model [67]. In our experimental conditions, LPS induced a complete block of ATP-mediated current potentiation in microglia, but did not affect basal Ca^2+^ levels or inhibit ATP-induced Ca^2+^ rise, indicating an uncoupling between [Ca^2+^]_i_ rise and current potentiation. Indeed, K_Ca_3.1 function was strongly reduced in LPS-treated cells, as revealed using the selective activator NS309 [68]. The absence of the Ca^2+^-evoked K^+^ current, along with the associated hyperpolarization, led to a reduced ATP-evoked Ca^2+^ entry, as shown by the faster Ca^2+^-transient kinetics observed in LPS-treated microglia. This last finding confirms a crucial role for Ca^2+^-activated K^+^ channels in human microglia, i.e., sustaining hyperpolarization to prolong Ca^2+^ entry [13]. The observation that LPS-treated microglial cells have a strongly reduced K_Ca_3.1 function is in contrast to a previous study, showing that LPS treatment did not change the K_Ca_3.1-mediated current in human microglia isolated from epileptic patients [3], in line with recent data showing a non-significant reduction of K_Ca_3.1 gene expression in microglia from LPS-injected mice [69]. We posit that this discrepancy could be explained by the different experimental settings, perforated patch vs. whole-cell configuration: if LPS treatment reduced the K_Ca_3.1 open probability through cytoplasmic diffusible factors, the resulting block of the Ca^2+^-mediated current potentiation would not be detectable in conventional whole-cell recording conditions where diffusible factors and second messengers have been dialyzed out. Microglia, in response to pro-inflammatory molecules such as LPS, depending on the duration and/or concentration of stimulus [70], may produce either more pro-inflammatory or anti-inflammatory (e.g., IL-4 or IL-10) molecules [71]. In turn, these molecules could promote different phosphorylation pathways involved in the modulation of the K_Ca_3.1 channel. An inhibitory effect of PKA on the human K_Ca_3.1 channel was recently shown to be due to channel phosphorylation on S334, with the consequent reduction of the CAM binding affinity for the channel [41, 65]. By contrast, K_Ca_3.1 channels are positively modulated by phosphorylation of H358 by nucleoside diphosphate kinase B [72].

What is the role of the ATP-evoked Ca^2+^-dependent modulation of human microglial K_Ca_3.1 channel in the context of epilepsy? Several reports indicated that the blockade of K_Ca_3.1 could represent a candidate approach for epilepsy treatment [60, 73], due to its ability to impair sustained Ca^2+^ entry in microglia during elevated neuronal activity and by shifting the phenotype of microglia towards an anti-inflammatory neuroprotective status [74, 75]. However, in a kindling model of temporal lobe epilepsy, K_Ca_3.1 blockade did not prevent seizures and even exacerbated pathology-related neuronal damage [20]. Besides, in a model of chronic epilepsy, a Ca^2+^-sensitive K_Ca_3.1-mediated component of the slow afterhyperpolarization of rat hippocampal pyramidal neurons has been reported to be downregulated by PKA [76]. Thus, future studies will be necessary to determine the role of K_Ca_3.1 activity in the context of epilepsy, also considering that different experimental models may underlay different microglial phenotypes.

## Conclusion

Our data confirm a crucial role of K_Ca_3.1 channel activity in microglial function in humans, linking ATP-induced Ca^2+^ signals to conductance changes, in a cellular process modulated by inflammatory stimuli. Future experiments will reveal whether microglial KCa3.1 could be a target to decrease the inflammatory mechanisms triggered by purinergic signals during epilepsy.

## Supplementary Information

**Additional file 1: Figure S1.** The activation of TRPC3/6 channels increases [Ca^2+^]_i_ and potentiates the transmembrane currents in human microglia. **Table S1.** Patients included in the study.

## Data Availability

The datasets used and/or analyzed during the current study are available from the corresponding author on reasonable request.

## Abbreviations

ANOVA: Analysis of variance
ATP: Adenosine three phosphate
[Ca^2+^]_i_: Free calcium concentration
CNS: Central nervous system
cGMP: Cyclic guanosine monophosphate
GFAP: Glial fibrillary acidic protein
Iba1: Ionized calcium-binding adapter molecule 1
IL-4, IL-10: Interleukin type 4, 10
K_Ca_3.1: Calcium-modulated potassium channel type 3.1
LPS: Lipopolysaccharide
PKA: Protein kinase type A
P2Y: Purinergic G protein-coupled receptors family
P2X4: Purinergic ionotropic receptor type 4
P2X7: Purinergic ionotropic receptor type 7
ROS: Reactive oxygen species
TRPC3/6: Transient receptor potential cation channels type 3/6
