# Supplementary material for: ATP-evoked intracellular Ca2+ transients shape the ionic permeability of human microglia from epileptic temporal cortex
Source: J Neuroinflammation. 2021 Feb 15;18:44. doi: 10.1186/s12974-021-02096-0 (PMC7883449; doi:10.1186/s12974-021-02096-0)
Supplement: Supplementary file 1 — Additional file 1: Figure S1. The activation of TRPC3/6 channels increases [Ca2+]i and potentiates the transmembrane currents in human microglia. Table S1. Patients included in the study. [file 12974_2021_2096_MOESM1_ESM.docx]

**Supplementary information**

*Figure S1. The activation of TRPC3/6 channels increases [Ca^2+^]_i_ and potentiates the transmembrane currents in human microglia.*

A, typical time-course of [Ca^2+^]_i_ changes elicited by application of GSK 1702934A, a selective agonist of TRPC3/6 channels (3 μM, 40 s, horizontal line) in a representative human microglial cell (patient #6). B, time course of the normalized mean current amplitude measured at -20 mV during GSK 1702934A application, averaged from 9 cells (2 from #6, 2 from #7 and 5 from #8). Voltage ramps were applied every 2s. Inset, histogram representing the mean current density at -20 mV before (grey) and at the end (white) of GSK 1702934A application. Black circles represent values from individual cells.

# Table S1. Patients included in the study

| Patient | Sex | Age at epilepsy onset (years) | Age at surgery (years) | Surgery | Histopathology |
| --- | --- | --- | --- | --- | --- |
| #1 | F | 25 | 29 | L-ATL | HS |
| #2 | F | 1 | 21 | L-ATL | HS |
| #3 | M | 21 | 44 | R-ATL | HS |
| #4 | M | 2 | 41 | L-ETL | HS |
| #5 | F | 21 | 41 | R-ATL | normal |
| #6 | M | 14 | 23 | Lesionectomy + R-ATL | DNET |
| #7 | M | 20 | 46 | R-ATL | HS |
| #8 | M | 15 | 26 | L-ATL | normal |
| #9 | M | 19 | 26 | R-ETL | FCDII A |
| #10 | M | 8 | 27 | L-ATL | HS |
| #11 | M | 14 | 50 | L-ATL | HS |
| #12 | F | 15 | 29 | L-ATL | HS |
| #13 | M | 25 | 27 | L-ATL | HS+ gangliocytoma |

# L: left, R: right, ETL: extended temporal lobectomy, ATL: anterior temporal lobectomy, HS: hippocampal sclerosis, DNET: Dysembryoplastic neuroepithelial tumour, FCD: focal cortical dysplasia.
